# King Edward's Hospital Fund

**Published:** 1907-12-21

**Authors:** 


### The Hospital

A JOURNAL OF

The Medical Sciences and Hospital Administration.

NEW SERIES. No. 39, VOL. II. [No. 1111, VOL. XLIII.] SATURDAY, DECEMBER 21, 1907.

### KING EDWARD'S HOSPITAL FUND.

On and from January 1, 1908, the affairs of this Fund will be conducted under the authority of the Act of Incorporation dated July 26, 1907. The passing of this Act constitutes an important development in the history of all voluntary hospitals. For although the King's Fund only deals strictly with the metropolitan hospitals and charities it has in truth had an important influence for good upon the economical administration of similar institutions throughout Great Britain. The King's Fund through its Distribution Committee makes grants each year to eligible hospitals within the area served by the Fund. The League of Mercy through its Distribution Committee makes grants to voluntary hospitals ministering to the needs of the inhabitants of the counties in which the League of Mercy organisation exists. Both the Fund and the League have elaborated a system of visitation, by which every participating hospital is inspected and reported upon each year by practitioners and laymen, and no grant is made unless the whole of the circumstances and needs of each institution dealt with are before the committee at the time the matter is considered. In this way the isolated position of many hospitals which formerly existed has been effectually removed, and to-day the Committee of every reputable hospital derives additional strength from the organisation of the King's Fund and the League of Mercy.

Founded originally by King Edward, when Prince of Wales, to help the voluntary hospitals of London, the King's Fund has proved a source of strength to hospital managers, not only in London, but in several important counties also. The authority of the Fund is undisputed. Its financial strength is so great as to be remarkable in the history of charity; its organisation has been gradually developed, and under the new Act it will now be perfected and placed upon a permanent basis. As now constituted the work of the Fund will in future be assigned to committees charged with authority to deal with the matters assigned to them. The Finance Committee will have the powers of the General Council with respect to all real and personal property possessed by the Fund, the investment of all moneys and funds available for that purpose, the management and control of the banking accounts, and of all stocks, funds or securities forming part of the Fund. The Distribution Committee possess the powers of the General Council to make rules and regulations to be observed with respect to applications from hospitals and institutions for grants, the determination as to which of such applications shall be considered, the making of inquiries into the circumstances and condition of institutions applying for grants, and the recommendation to the President and General Council as to the distribution of the funds of the Corporation. The Convalescent Homes Committee will fulfil identical duties in regard to convalescent homes, and certain sanatoria. Finally the powers of the General Council in respect of all other matters, except the expenditure of funds beyond £3,500 and the making of rules and regulations under section 8 of the Act, will be vested in a Committee, heretofore known as the Executive Committee. An interesting discussion was raised by Sir William Collins as to the name of this Committee, which is to remain the "Executive Committee" for one year, when the name will be reconsidered and finally determined. Sir William Collins did good service in raising this point, and in securing an instruction to this Committee to inquire and report whether in their opinion rules and regulations for the general conduct of the business of the Fund should be formulated or not.

The Prince of Wales in two excellent speeches, which are reported in another column, lucidly explained the present position of the Fund, and notified certain important changes and proposed extensions. He expressed the wish that pecuniary support should be given to sanatoria for tuberculous cases, but, as Mr. John Burns the President of the Local Government Board, pointed out, this is a matter which affects all classes of people, and must therefore involve an expenditure far and away beyond the resources of the King's Fund. Besides, the making of provision through sanatoria is in reality the business of the Government and local authorities through their public health departments. We hope, therefore, that the King's Fund may be retained mainly for the support of the voluntary hospitals in accordance with the original constitution of the Fund, any help extended to sanatoria being confined to convalescent institutions attached to hospitals which already receive grants.

The Prince paid a well-deserved tribute to Mr.

Danvers Power on his retirement from the office of honorary secretary. Mr. Power has done important service in laying down the lines on which the expenditure tables and other statistics are to be made by the hospitals. His reports have had a marked effect in improving the economical administration of hospitals, and the zeal and energy he has put into his labours during his four years of office have been of practical value to the development and usefulness of the King's Fund. The new honorary secretary Mr. Frederick M. Fry has been associated with the Fund for some years, and has devoted a great deal of time to the work. He is a careful and zealous worker and his appointment has given great satisfaction to all interested in the King's Fund.

